# Evolutionarily Conserved *nodE, nodO*, T1SS, and Hydrogenase System in Rhizobia of *Astragalus membranaceus* and *Caragana intermedia*

**DOI:** 10.3389/fmicb.2017.02282

**Published:** 2017-11-20

**Authors:** Hui Yan, Jian Bo Xie, Zhao Jun Ji, Na Yuan, Chang Fu Tian, Shou Kun Ji, Zhong Yu Wu, Liang Zhong, Wen Xin Chen, Zheng Lin Du, En Tao Wang, Wen Feng Chen

**Affiliations:** ^1^State Key Laboratory of Agro-Biotechnology, College of Biological Sciences and Rhizobium Research Center, China Agricultural University, Beijing, China; ^2^State Key Laboratory of Animal Nutrition, Beijing Engineering Technology Research Center of Raw Milk Quality and Safety Control, College of Animal Science and Technology, China Agricultural University, Beijing, China; ^3^National Engineering Laboratory for Tree Breeding, College of Biological Sciences and Technology, Beijing Forestry University, Beijing, China; ^4^Beijing Institute of Genomics, Chinese Academy of Sciences, Beijing, China; ^5^Departamento de Microbiología, Escuela Nacional de Ciencias Biológicas, Instituto Politécnico Nacional, Mexico, Mexico

**Keywords:** *nodE*, *nodO*, T1SS, hydrogenase system, *Mesorhizobium*, symbiotic specificity, *Astragalus*, *Caragana*

## Abstract

*Mesorhizobium* species are the main microsymbionts associated with the medicinal or sand-fixation plants *Astragalus membranaceus* and *Caragana intermedia* (AC) in temperate regions of China, while all the *Mesorhizobium* strains isolated from each of these plants could nodulate both of them. However, *Rhizobium yanglingense* strain CCBAU01603 could nodulate AC plants and it's a high efficiency symbiotic and competitive strain with *Caragana*. Therefore, the common features shared by these symbiotic rhizobia in genera of *Mesorhizobium* and *Rhizobium* still remained undiscovered. In order to study the genomic background influencing the host preference of these AC symbiotic strains, the whole genomes of two (*M. silamurunense* CCBAU01550, *M. silamurunense* CCBAU45272) and five representative strains (*M. septentrionale* CCBAU01583, *M. amorphae* CCBAU01570, *M. caraganae* CCBAU01502, *M. temperatum* CCBAU01399, and *R. yanglingense* CCBAU01603) originally isolated from AC plants were sequenced, respectively. As results, type III secretion systems (T3SS) of AC rhizobia evolved in an irregular pattern, while an evolutionarily specific region including *nodE, nodO*, T1SS, and a hydrogenase system was detected to be conserved in all these AC rhizobia. Moreover, *nodO* was verified to be prevalently distributed in other AC rhizobia and was presumed as a factor affecting the nodule formation process. In conclusion, this research interpreted the multifactorial features of the AC rhizobia that may be associated with their host specificity at cross-nodulation group, including *nodE, nodZ*, T1SS as the possible main determinants; and *nodO*, hydrogenase system, and T3SS as factors regulating the bacteroid formation or nitrogen fixation efficiency.

## Introduction

Based on their tremendously important medicinal values and remarkable sand-fixing effects, *Astragalus* and *Caragana* species (belonging to Tribe Galegeae and Tribe Hydesareae, respectively) are widely cultivated in the northwest region of China. The diversity of rhizobia associated with these plants has been extensively studied (Zhao et al., 2008; Li et al., 2012; Yan et al., 2016). Previous researches revealed that both the cultivated and wild AC plants mainly nodulate with *Mesorhizobium* strains, while strains from other rhizobial genera occupied minor proportion in the nodules (Zhao et al., 2008; Lu et al., 2009; Li et al., 2012; Yan et al., 2016). However, an exception was found in a previous study in our laboratory that strain *R. yanglingense* CCBAU01603 has a more competitive nodulation ability than the representatives of *Mesorhizobium* species (*M. silamurunense* CCBAU01550, *M. silamurunense* CCBAU45272, *M. septentrionale* CCBAU01583, *M. amorphae* CCBAU01570, *M. caraganae* CCBAU01502) when *Caragana* plants were grown in sterile vermiculite (Ji et al., 2017). From these results, it could be seen that the nodulation specificity and competence of rhizobial strains on AC plants was different among different strains. However, the genetic characteristics that influence the nodulation phenotype of AC rhizobia is still unknown.

As reported in previous studies, the nodulation ability of rhizobia was determined by the symbiosis genes located on the symbiosis island/plasmid (Hirsch et al., 2001) and type III secretion system (Okazaki et al., 2013). Studies on recognition and symbiotic specificity between legume hosts and rhizobia have made deep significance for understanding the interaction between the host plants and the microsymbionts. Currently, with the development of next-generation sequencing technology, the symbiotic characteristics of rhizobia are facing further studies of genomic analysis. Some whole genomes of strains within the genera *Sinorhizobium* and *Bradyrhizobium* that nodulate *Glycine max* (Tian et al., 2012) or *Medicago* (Sugawara et al., 2013) have been studied for their host specificity. However, the study of host specificity referring to the rhizobia that comprises two genera and associated with different hosts in a cross-nodulation group is still deficient. Considering the great morphology and phylogenetic differences between AC plants, the AC rhizobia might be valuable candidates for further research to dig out the symbiotic specificity at the level of cross-nodulation group.

The major symbiotic rhizobia nodulating AC hosts were *Mesorhizobium* strains (Lu et al., 2009; Li et al., 2012; Yan et al., 2016) harboring conserved *nodC* genes differed from those in non-AC-nodulating strains, while the *nodC* of AC symbiotic strain *R. yanglingense* CCBAU01603 was not clustered closely to the AC-nodulating *Mesorhizobium* strains. Since *nodC* gene is an important determinant of chitin oligosaccharide chain of Nod factor that is a contributor to host specificity according to the study on *Sinorhizobium* (former *Rhizobium*) *meliloti* (Kamst et al., 1997), the same preference of nodulation with AC plants by *R. yanglingense* CCBAU01603 and the *Mesorhizobium* strains might be determined by other symbiotic genes. Therefore, in order to better understand the mutual genomic characteristics of *Mesorhizobium* and *Rhizobium* strains that preferred AC plants at genus level, 7 rhizobia were whole-genome sequenced and analyzed for the nodulation genes, secretion systems and evolutionarily specific gene clusters.

## Materials and methods

### Strains and their growth conditions

*M. silamurunense* CCBAU01550 and *M. silamurunense* CCBAU45272 originally isolated from *Astragalus membranaceus* (Zhao et al., 2008), and *M. temperatum* CCBAU01399 (Lu et al., 2009), *M. amorphae* CCBAU01570 (Li et al., 2012), *M. septentrionale* CCBAU01583 (Li et al., 2012), *M. caraganae* CCBAU01502 (Guan et al., 2008), and *R. yanglingense* CCBAU01603 (Li et al., 2012) originally isolated from *Caragana intermedia* were used for whole genome sequencing in this study. Nodulation tests performed in our laboratory further confirmed the nodulation ability of these strains with AC hosts. All these rhizobial strains grown on TY agar at 28°C for 3–5 days, and cultivated in TY broth at 28°C at 180 rpm for 3 days.

### DNA isolation

Cell pellets were collected from 1 mL bacterial cultures by centrifugation at 13,000 rpm for 2 min. Then the cell pellets were resuspended in 1 mL sterile water for washing then centrifuged. The genomic DNA was extracted from the pellets using Wizard® Genomic DNA Purification Kit (Promega) according to the suggested procedure. Extracted DNA were temporarily maintained at −80°C before being sent to the sequencing companies (BGI-Shenzhen, China).

### Genome sequencing, assembly, and annotation

The genomes were sequenced on platform Illumina HiSeq 2000 (100× sequencing depth) by BGI-Shenzhen China, and low-quality sequencing reads were filtered. SOAPdenovo v2.01 in Linux operating system (http://soap.genomics.org.cn/soapdenovo.html) was used to assemble the genomes and Gapcloser v1.12 was used to close the gaps after assembly. Different Kmer values were tested from 17 to 91 (odd numbers) and the assembly results with the highest N50 values were reserved as the best assembly result (Luo et al., 2012). The whole genome sequences of the seven strains have been deposited in GenBank (Table 1). Glimmer v3.02 (Delcher, 2006) that was widely used in bacteria gene prediction (Delcher et al., 2007) was used to predict coding genes based upon interpolated Markov models (http://ccb.jhu.edu/software/glimmer/index.shtml; Salzberg et al., 1998). Sequences of predicted genes and proteins were extracted using Perl scripts. Then protein sequences were aligned against the non-redundant (nr) protein database of NCBI for annotation.

**Table 1:** The general genome information of the genomes used in this study.

**Group**	**Strains**	**Host**	**GenBank biosample No**.	**Genome size (Mb)**	**(G+C) mol%**	**tRNA genes**	**Protein-coding sequences (CDSs)**
AC rhizobia	*R. yanglingense* CCBAU01603^*^	*C. intermedia*	SAMN02584782	7.67	59.0	70	8,256
	*M. silamurunense* CCBAU01550^*^	*A. membranaceus*	SAMN02712003	7.07	62.7	49	7,496
	*M. silamurunense* CCBAU45272^*^	*A. membranaceus*	SAMN04278952	7.22	63.1	49	7,692
	*M. septentrionale* CCBAU01583^*^	*C. intermedia*	SAMN02584783	7.67	62.1	51	7,356
	*M. amorphae* CCBAU01570^*^	*C. intermedia*	SAMN02712002	7.37	61.2	48	7,141
	*M. caraganae* CCBAU01502^*^	*C. intermedia*	SAMN02585731	7.20	62.4	50	7,005
	*M. temperatum* CCBAU01399^*^	*C. intermedia*	SAMN02585732	7.42	62.4	62	8,604
Other hosts	*M. metallidurans* STM2683	*Anthyllis vulneraria*	SAMEA2272539	6.23	62.0	45	5,962
	*M. ciceri* bv. *biserrulae* WSM1271	*Biserrula pelecinus*	SAMN00713576	6.70	61.7	49	6,264
	*M. ciceri* ca181	*Cicer arietinum*	SAMN02470606	6.42	61.5	56	6,694
	*M. loti* MAFF303099	*Lotus japonicus*	SAMD00061086	7.60	60.6	54	7,281
	*Mesorhizobium* sp. WSM1293	*Lotus* sp.	SAMN02597200	6.94	61.8	52	6,706
	*M. loti* R88b	*Lotus corniculatus*	SAMN02597285	7.20	62.4	54	7,179
	*M. opportunistum* WSM2075	*Biserrula pelecinus*	SAMN00713576	6.88	62.9	53	6,508
	*M. ciceri* WSM4083	*Bituminaria bitumosa*	SAMN02584909	6.84	61.3	50	6,616
	*R. etli* CFN42	*Phaseolus vulgaris*	SAMN02603106	6.53	60.5	50	6,156
	*Rhizobium* sp. BR816	*Leucaena leucosephala*	SAMN02261311	6.95	60.4	55	6,752
	*R. etli* bv*. mimosae* Mim1	*Mimosa affinis*	SAMN02603105	7.20	60.4	51	6,792
	*R. leguminosarum* bv*. trifolii* WSM2304	*Trifolium polymorphum*	SAMN00000679	5.87	60.6	53	6,415
	*Rhizobium* sp. IRBG74	*Sesbania cannabina*	SAMEA3138824	5.46	58.7	54	5,478
	*R. leguminosarum* bv*. phaseoli* 4292	*Phaseolus vulgaris*	SAMN02261312	7.35	60.2	50	7,177
Outgroup	*B. japonicum* USDA6	*Glycine max*	SAMD00060992	9.21	63.7	57	8,409
	*Cupriavidus taiwanensis* LMG19424	*Mimosa pudica*	SAMEA3138280	6.48	65.03	63	5,654

* *Strains that sequenced in this study*.

### Bioinformatics analysis

In this analysis, the whole genome sequences acquired in the present study and those of other 14 *Mesorhizobium* and *Rhizobium* strains downloaded from the NCBI database were included (see Table 1 for detail). Genome sequences of *B. japonicum* USDA6 and *Cupriavidus taiwanensis* LMG19424 were also included as out group in the phylogenetic analysis. PGAP (Pan-Genome Analysis Pipeline) was used to analyze the pan-genome and core-genome of tested strains (Zhao et al., 2012). Specific genes of each group were extracted using Perl script according to the results of PGAP analysis.

All these strains, except *R. yanglingense* CCBAU01603 and the out group strains, were divided into 3 groups: AC-originating *Mesorhizobium* (ACiM, 6 strains), non-AC-originating *Mesorhizobium* (non-ACiM, 8 strains), and non-AC-originating *Rhizobium* (non-ACiR, 6 strains). To identify the phylogenetically conserved genes present in AC rhizobia (including ACiM and *R. yanglingense* CCBAU01603), 20 tested strains of three groups (ACiM, non-ACiM, non-ACiR) were aligned to the 8256 proteins of *R. yanglingense* CCBAU01603 using phmmer program in HMMER3.0 package (Eddy, 2011). Bit score of each gene was analyzed among the three groups using ANOVA in R software based on the FDR adjusted *p*-value with a cutoff value of 0.001, then the evolutionarily specific genes for AC rhizobia were identified according to the identity bit score and *p*-value. Alignment of homologous genes was performed with ClustalW2.0 (Larkin et al., 2007), and phylogenetic analysis was operated with PhyML3.0 (Guindon et al., 2010) and SplitsTree (Huson and Bryant, 2006). The similarity of homologous genes was calculated using MEGA 6.06 software (Tamura et al., 2013) with p-distance method.

### PCR universal verification for *nodO* genes

To verify whether *nodO* gene was universal in other common AC rhizobia, PCR verification was performed using primers *nodO*-131F (5′-GCGAGGGCAGTGACCAA-3′) and *nodO*-554R (5′-GCCGCACCGCTGTAGAA-3′). This pair of primers was designed according to the *nodO* sequence of strain CCBAU45272 using the primer premier 5.0 software (Lalitha, 2000). The PCR reaction system was 50 μL, including Taq Mix (23 μL), 131F (1 μL), 554R (1 μL), template DNA (1 μL), and ddH_2_O (24 μL). The PCR protocol was 95°C (5 min), 30 cycles [94°C (50 s); 56°C (50 s); 71°C (50 s)], and 72°C (5 min).

### Construction of *nodO* mutant of CCBAU01603

Upstream fragment (727 bp) of the target gene was amplified using primers *nodO* up F:EcoRI-5′-CGGAATTC-GGCAAACATTTACCGACCGACTA-3′ and *nodO* up R:KpnI-5′-GGGGTACC-CTTTACCATCGCAAACACTCCT-3′. Downstream fragment (812 bp) of the target gene was amplified using primers: *nodO* down F:snaBI-5′-GACTTACGTA-AGTTCGTTCACCTGAGCGG-3′ and *nodO* down R:sacI-5′-GACGAGCTC-CGCGTTGAAAGCGGAAG-3′. The PCR system and protocol was the same as mentioned above of *nodO* universal fragment. After amplification, the empty vector plasmid pRL1063a (Wolk et al., 1991) and upstream fragment were digested with double endonuclease restriction (*Eco*RI and *Kpn*I) for 4 h, respectively. Double digested plasmids and upstream fragment were linked by T4 DNA ligase at 16°C for 8 h. The upstream fragment containing plasmid was transformed into strain *E. coli* DH5α using heat shock method, then the integrated DH5α strain was incubated on LB plating medium for 24 h. Several single colony isolates were inoculated in TY broth in 37°C at 180 rpm for 8 h, and then the strains were delivered to sequencing company to screen the isolate that without nucleotide mutations. The same method was used to link the downstream fragment to plasmid pRL1063a. Then the donor bacteria that carried vector containing upstream fragment and downstream fragment has been constructed.

Homologous pair exchange method was used to construct *nodO* deletion mutant of strain CCBAU01603. Donor strain, accessory strain and receptor strain were fully mixed according to ratio of 3:2:10, and the mixture was spread on TY plates to incubate at 28°C for 24–48 h. Then the cultures were scraped into 1 mL of physiological saline and diluted to 10^−6^–10^−8^. Aliquot of 0.1 mL of the last three dilutions was spread on plates with TY medium. After incubated for 48 h at 28°C, single colony isolates were picked out and inoculated on TY medium supplied with 5 μg mL^−1^ tetracycline for further incubation at 28°C for another 48 h. Single colony isolates lacking the resistance to tetracycline were obtained. Then the *nodO* deletion mutants were verified by absence of *nodO* amplification with the methods mentioned above.

### Inoculation test and the observation of bacteroids

Wild type and *nodO* deletion mutant of strain CCBAU01603 were cultured in 5 mL of TY broth at 28°C for 2 days and were inoculated on hosts *A. membranaceus, C. intermedia*, and the promiscuous legume *Sophora flavescens* separately using the protocol described previously (Yan et al., 2016). The plants were cultivated in vermiculite for 40 days in greenhouse with a cycle of 16 h light at 25°C and 8 h dark at 22°C. Five replicates were repeated. Then the nodules of wild type and *nodO* mutant were obtained and fixed in 2.5% glutaraldehyde solution immediately. The fixed nodules were delivered to the Electron Microscope Lab to do further technological process, then the bacteroids were observed at 2,500× and 10,000× magnification using transmission electron microscope in China Agriculture University.

## Results

### The general features of the genomes used in this study

As results (Table 1), the genome sizes of AC-originating strains were about 7.07–7.67 Mb, which were generally larger than those of non-AC-originating strains that presented genome sizes 6.23–7.60 Mb (*t*-test *p*-value = 0.0116). Likewise, the CDS numbers present a good correlation with the genome size (*R*^2^ = 0.979), and the number of predicted CDSs of AC-originating strains were obviously higher than that of the non-AC-originating strains. Besides, the DNA G+C contents of ACiM strains (61.2–63.1%) were significantly higher than those of the non-AC-originating strains (60.2–62.0%; *t*-test, *p*-value = 0.02), excepts that DNA G+C content of *R. yanglingense* CCBAU01603 was 59.0 mol%.

**Figure 1:**
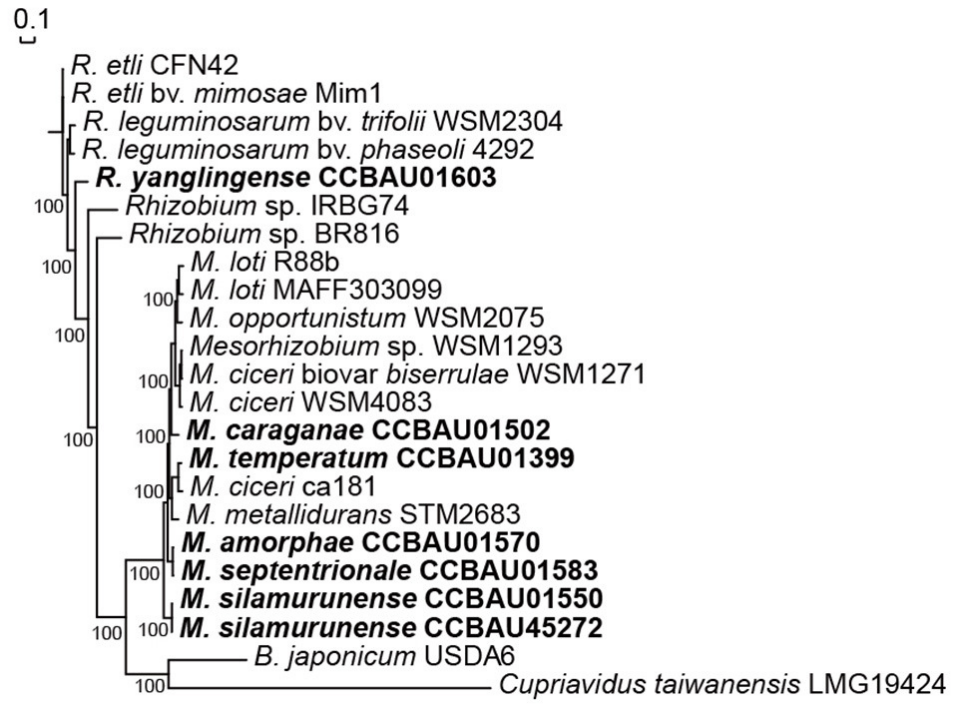
Phylogenetic relationships of AC-isolated and non-AC-isolated strains based on 362 single copy core genes. Maximum likelihood (ML) method was used to construct the tree using PhyML3.0. Total 362 single copy core genes were exacted according to the PGAP results and connected after alignment using clustalW2.0. Bold represents the AC rhizobia that sequenced in this research.

Our analysis revealed that the 21 researched rhizobia (14 *Mesorhizobium* and 7 *Rhizobium*) strains contained a pan-genome of 29,274 putative protein-coding genes. Moreover, the 21 genomes shared a core genome of 823 genes, accounting for 9.6–15.0% of whole genomes, representing a set of conserved orthologous genes for both *Mesorhizobium* and *Rhizobium* genera. Out of these 823 core genes, 362 single copy core genes that represents the reliable genetic relationship of 21 rhizobia were used to perform a maximum likelihood phylogenetic analysis, and results showed that rhizobia of *Mesorhizobium* and *Rhizobium* evolved divergently as two different genera (Figure 1). It's true that *R. yanglingense* CCBAU01603 clustered with other *Rhizobium* strains, and all the *Mesorhizobium* strains were phylogenetically clustered in a conserved branch.

### Conserved genes in AC-originating strains

**Figure 2:**
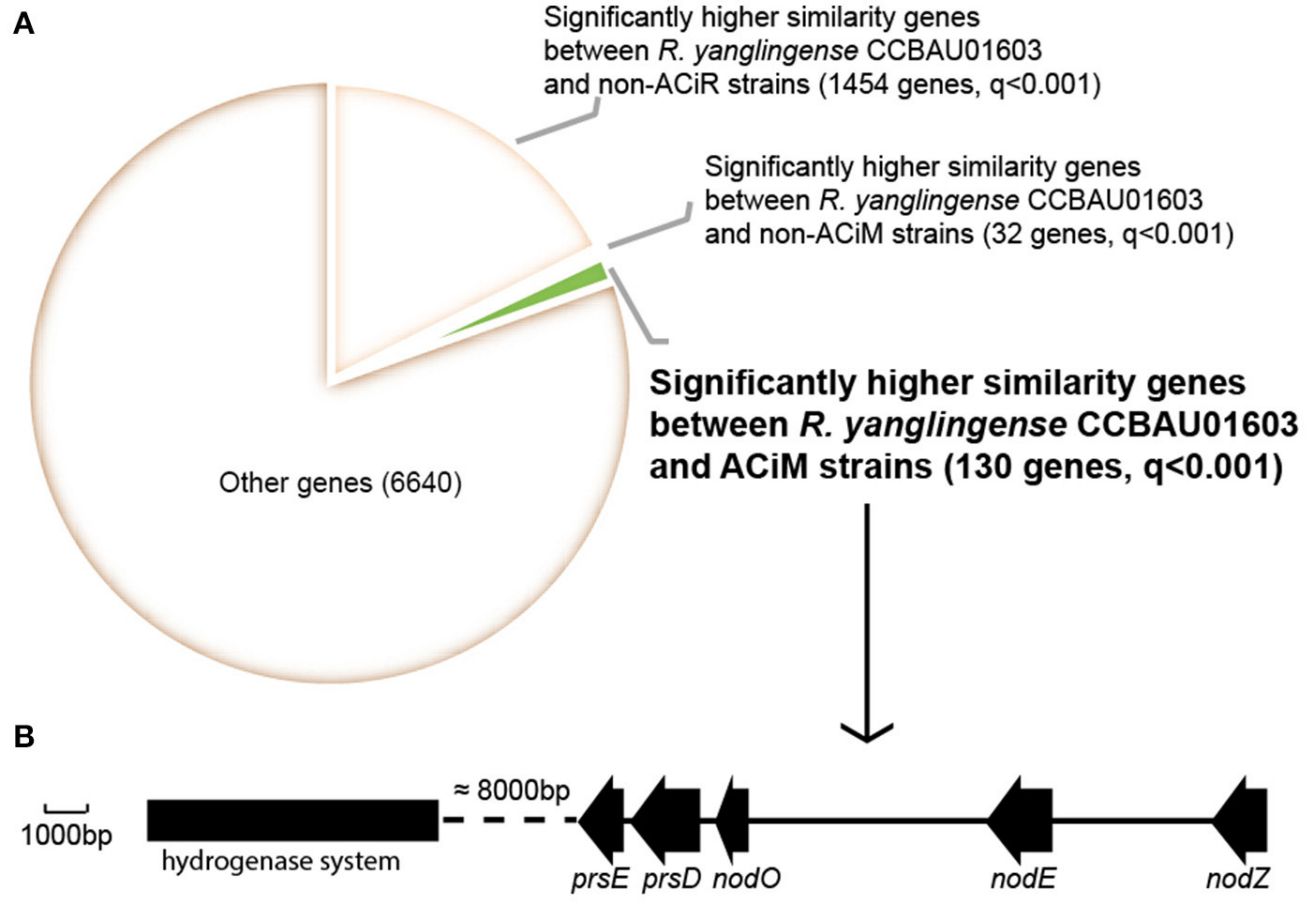
Analysis of evolutionarily closer genes in strains of these three groups (ACiM, non-ACiM, and non-ACiR) in comparison to *R. yanglingense* CCBAU01603. **(A)** Evolutionarily closer genes that shared higher genetic similarities in comparison with *R. yanglingense* CCBAU01603. Data in parentheses represent numbers of shared evolutionarily closer genes. Bold was evolutionarily closer genes for AC rhizobia including two genera (*Mesorhizobium* and *Rhizobium*). *q-*value threshold was 0.001. **(B)** Arrangement of evolutionarily closer genes between ACiM and *R. yanglingense* CCBAU01603, including evolutionarily specific *nodE, nodO*, T1SS and hydrogenase system. Dash line was a region of about 8 kb.

Using the program phmmer in HMMER3.0 that based on the alignment of protein conserved domain (Eddy, 2011), a set of 130 genes that conserved for ACiM strains and *R. yanglingense* CCBAU01603 were screened out, which had significantly higher similarities between ACiM and strain CCBAU01603 than non-ACiM and non-ACiR groups, and were presumed as homologous genes that have closer evolutional relationships between ACiM group strains and *R. yanglingense* CCBAU01603, and may be responsible for nodulation specificity preference (based on FDR value <0.001; Figure 2A). Interestingly, these 130 genes derived from 5 scaffolds, out of which, a scaffold contains the nodulation island including evolutionarily specific *nodZ, nodE, nodO*, and T1SS for all these seven AC-originating strains (Figure 2B).

### Conserved nodulation genes in AC-originating strains

**Figure 3:**
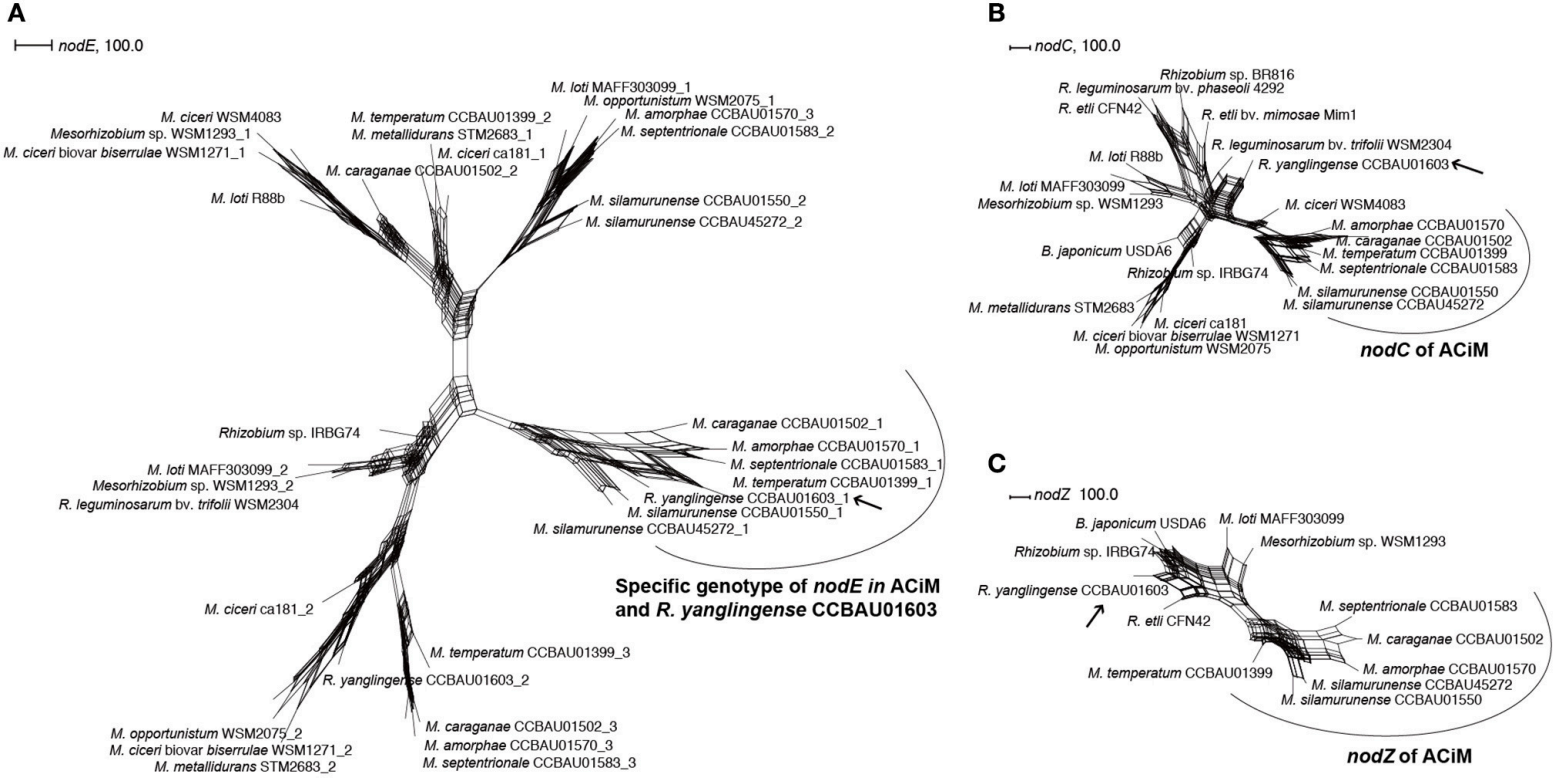
Phylogenetic trees of *nodE* **(A)**, *nodC* **(B)**, and *nodZ* **(C)** genes based on network construction using SplitsTree 4.13.1. The genes were aligned using clustalW2.0. Phylogenetic networks were constructed using SplitsTree 4.13.1. The arrow points to strain *R. yanglingense* CCBAU01603, which was the unique *Rhizobium* strain that isolated from AC plants. And the curve covers the ACiM strains.

Since nodulation genes are the key determinants in host recognition and signal transduction in rhizobia, some nodulation genes were comparatively studied among the AC microsymbionts. *R. yanglingense* CCBAU01603 and other 6 ACiM strains shared the highly evolutionarily conserved *nodE*, even multiple copies of *nodE* were detected in *R. yanglingense* CCBAU01603 (Figure 3A). Besides, *nodC* genes of ACiM strains were phylogenetically distinctive from those of other non AC rhizobia, and also divergent from that of *R. yanglingense* CCBAU01603 (Figure 3B). Even though, the *nodC* of *R. yanglingense* CCBAU01603 shared relatively higher similarity with ACiM (p-distance of NodC: 0.181–0.194) compared to non-ACiM and non-ACiR groups (p-distance of NodC: 0.200–0.308). In addition, an uncommon gene *nodZ* was identified in all the 7 AC-originating strains, but it evolved divergently in *R. yanglingense* CCBAU01603 compared with those in the six AC-originating *Mesorhizobium* strains (Figure 3C).

### Conserved hydrogenase systems and widely spread *nodO* genes in AC-originating strains

**Figure 4:**
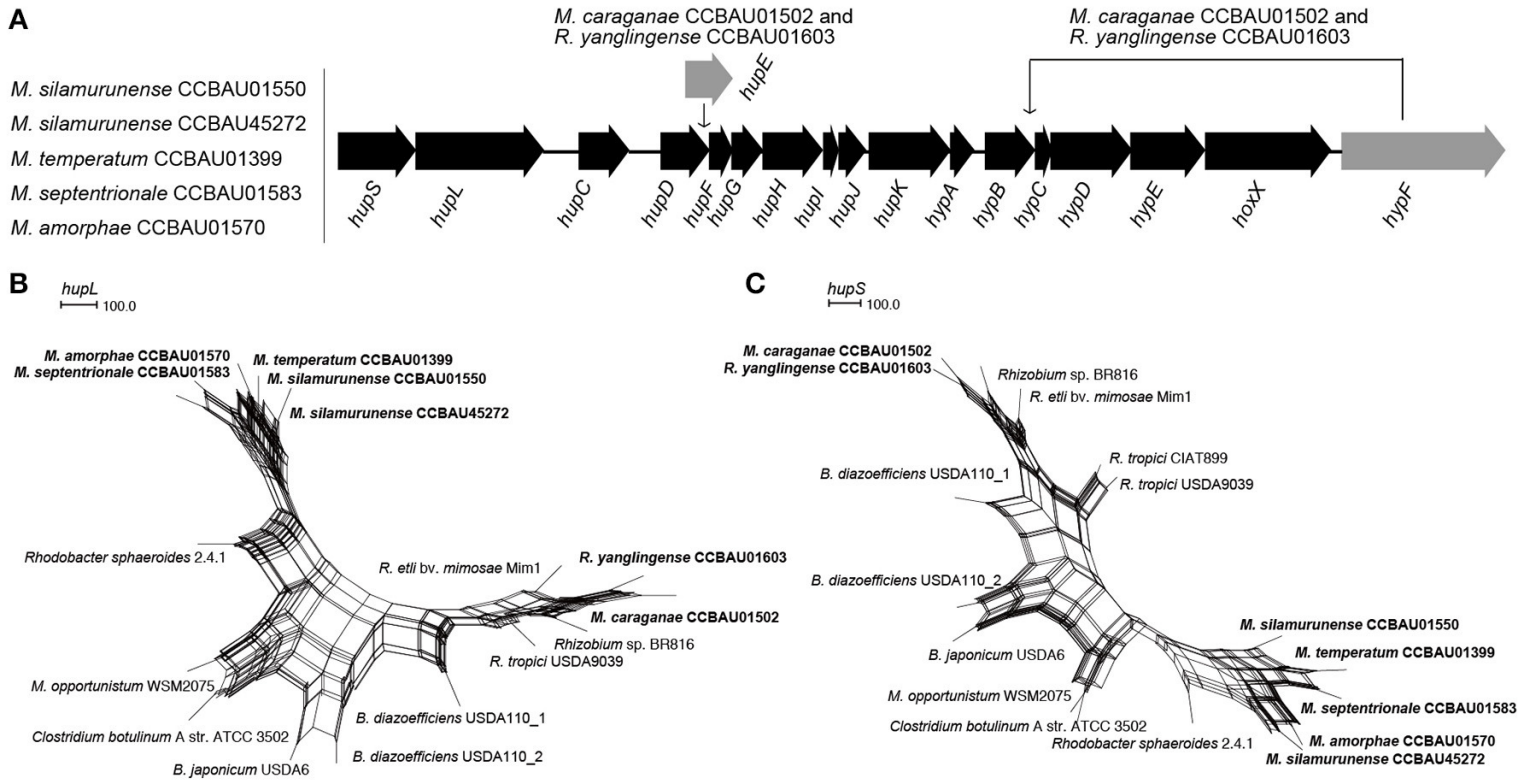
Arrangement of hydrogenase system gene clusters **(A)** and phylogenetic relationships of *hupL* **(B)** and *hupS* **(C)** of the AC-isolated (Bold) and other strains (Regular). **(A)** These genes arranged from *hupS* to *hypF* without *hupE* for strains CCBAU01550, CCBAU45272, CCBAU01399, CCBAU01583, and CCBAU01570. A *hupE* gene was inserted between *hupD* and *hupF*, and *hypF* translocated for strains CCBAU01502 and CCBAU01603. **(B,C)** The genes were aligned using clustalW2.0. Phylogenetic networks were built using SplitsTree 4.13.1.

According to the results of phmmer alignment analysis, a system that responsible for hydrogenase biosynthesis (*hup*) was detected in the 7 AC-originating strains (Figure 4A), which was supposed to be positively related to the nitrogen fixation efficiency in some rhizobial strains. In the other 14 reference strains, hydrogenase systems only have been detected in *Rhizobium* sp. BR816, *R. etli* bv. *mimosae* Mim1, and *M. opportunistum* WSM2075. Interestingly, the hydrogenase systems were found to be evolved from two ancestries in these 7 AC-originating strains: *M. caraganae* CCBAU01502 and *R. yanglingense* CCBAU01603 shared a mutual origin (p-distance: 0.010), which presented closer genetic distance from the other non-AC-originating strains (p-distance: 0.050–0.101) than the other five ACiM strains (p-distance: 0.193–0.200; Figures 4B,C).

**Figure 5:**
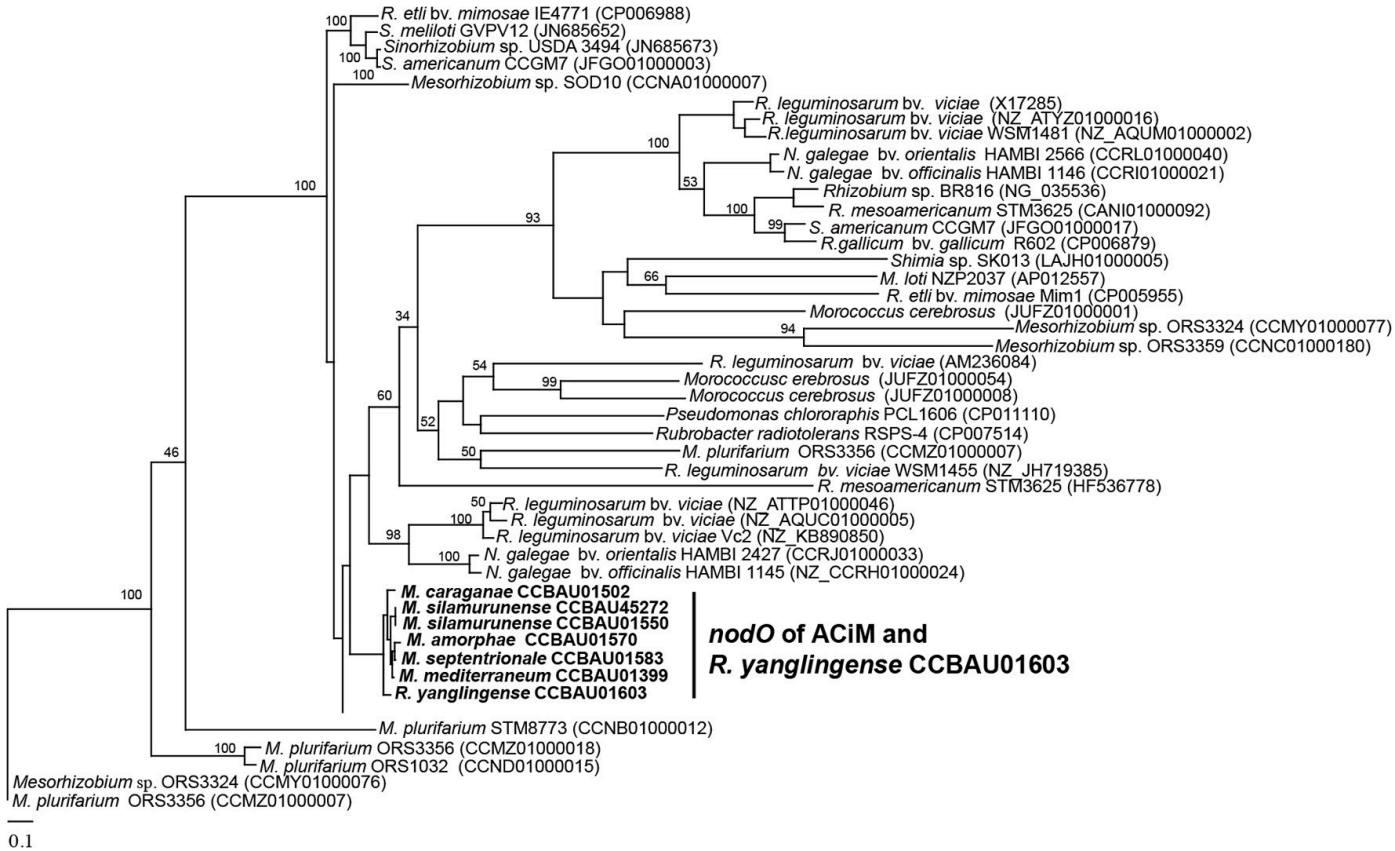
Phylogenetic tree of *nodO* genes based on Maximum Likelihood (ML) method. The genes were aligned using clustalW2.0. Phylogenetic tree was constructed using PhyML3.0 with bootstrap value of 100. Bold strains represent specific type of *nodO* for AC-originating rhizobia (ACiM and *R. yanglingense* CCBAU01603). Numbers in parentheses represent the gene accession number in NCBI.

Moreover, specifically conserved *nodO* as a host range expanded gene was also detected in all the seven AC-originating strains. Since *nodO* also presented in the broad host strains *Rhizobium* sp. BR816 and *R. etli* bv. *mimosae* Mim1, it may contribute to the broad host range phenotype in rhizobial strains. Therefore, more *nodO* sequences were downloaded from GenBank database to analyze their phylogenetic relationships with those AC-originating strains. As shown in Figure 5, the *nodO* genes of the 7 AC-originating strains were obviously divergent from those of the other rhizobia, and *R. yanglingense* CCBAU01603 harbored a *nodO* gene that shared extremely high similarity (95.6–98.8% nucleotide similarity) with the 6 ACiM strains. In addition, the *nodO* genes of AC-originating rhizobia shared only 46.7–75.8% similarities with those in other rhizobia. Moreover, *nodO* was exactly amplified out in all 14 representative strains (with nodulation ability) of 367 isolates from *Astragalus* grown in 3 provinces of China (Yan et al., 2016) using universal primers of *nodO* (Table S1).

### The T3SS and conserved T1SS of AC-originating rhizobia

**Figure 6:**
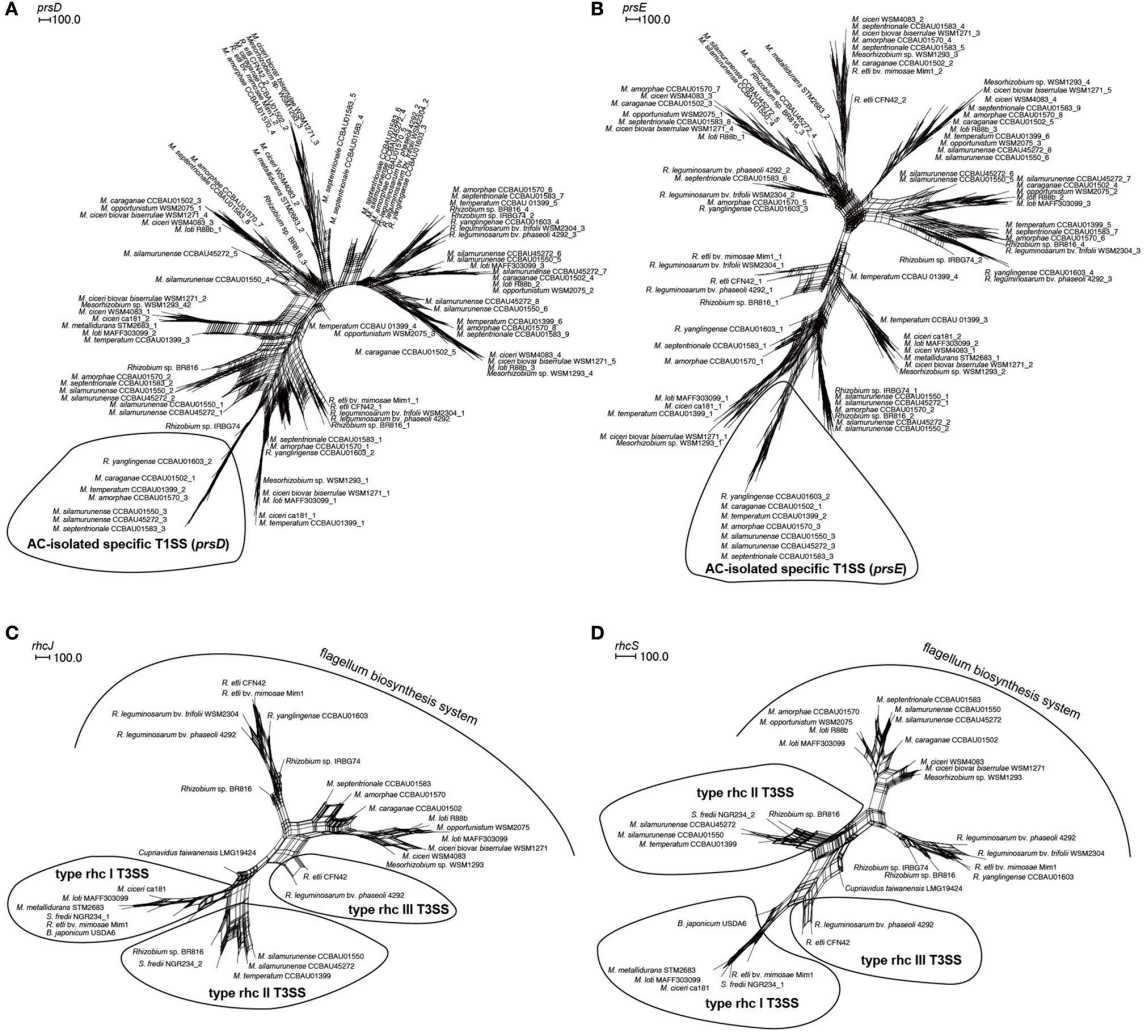
Phylogenetic trees of T1SS (*prsD* and *prsE*) and reserved T3SS genes (*rhcJ* and *rhcS*) based on SplitsTree network. The genes were detected using phmmer in HMMER3 with *E*-value and coverage were 1e-5 and 50%, respectively. The genes were aligned using clustalW2.0. Maximum Likelihood (ML) phylogenetic networks were constructed using SplitsTree 4.13.1. **(A)** *prsD* (T1SS); **(B)** *prsE* (T1SS); **(C)** *rhcJ* (T3SS); **(D)** *rhcS* (T3SS).

For the strains studied in this research, 4–8 pairs of *prsD*/*prsE* genes (T1SS structural genes) were identified in these 7 AC-originating strains, but only 2–4 pairs were discovered in the other strains. Out of which, a pair of evolutionarily conserved and private *prsD*/*prsE* genes were detected right at the downstream of *nodE* and *nodO* genes and the upstream of *hup* biosynthesis system gene cluster. And this pair of *prsD*/*prsE* genes in the 7 AC-originating strains evolved distinctively from non-ACiM and non-ACiR rhizobia, which indicated a homologous and consistent relationship with its adjacent *nodE* and *nodO* genes (Figures 6A,B).

However, typical T3SS was identified for strains *M. silamurunense* CCBAU01550, *M. silamurunense* CCBAU45272, and *M. temperatum* CCBAU01399, and in the phylogenetic trees of *rhcJ* and *rhcS* genes (structural genes of T3SS), these three strains were grouped together with type rhc-II T3SS of *Rhizobium* sp. BR816 and *Sinorhizobium fredii* NGR234. While the remaining AC-rhizobia harbored atypical T3SSs (more similar to flagellum biosynthesis system) and clustered with other *Mesorhizobium* or *Rhizobium*, according to their genus affiliation (Figures 6C,D).

### The nodulation test of CCBAU01603 wild type strain and its *nodO* mutant

**Figure 7:**
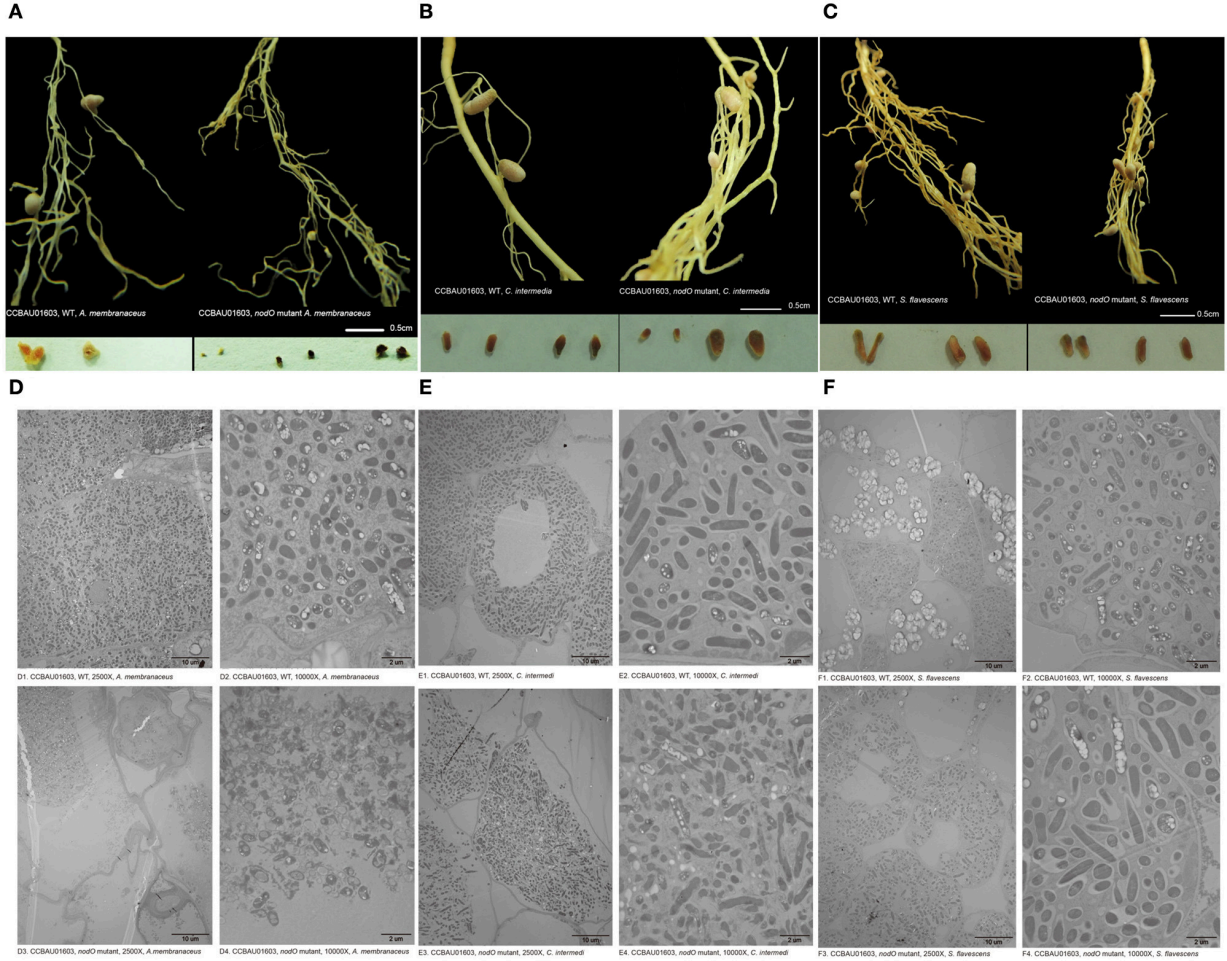
Nodulation phonotype and bacteroids morphology of *nodO* mutant of *R. yanglingense* CCBAU01603 under transmission electron microscope (TEM). **(A–C)** Nodulation phonotype on *A. membranaceus* **(A)**, *C. intermedia* **(B)**, and *S. flavescens* **(C)**, respectively. **(D–F)** Bacteroids morphology of strains inoculated to *A. membranaceus* **(D)**, *C. intermedia* **(E)**, and *S. flavescens* **(F)**, respectively. D1, D2, E1, E2, F1, F2: CCBAU01603 wild type. D3, D4, E3, E4, F3, F4: CCBAU01603 *nodO* mutant. D1, D3, E1, E3, F1, F3: 2,500×. D2, D4, E2, E4, F2, F4: 10,000×. WT: wild type.

A *nodO* deletion mutant of CCBAU01603 was constructed to verify the impact of *nodO* on symbiosis. The wild type of CCBAU01603 could form effective nodules on *A. membranaceus, C. intermedia*, and *S. flavescens*. But, the *nodO* deletion mutant formed ineffective small nodules on *A. membranaceus*, which present white or black nodule sections (Figure 7A). However, the nodules formed by mutant of CCBAU01603 on *C. intermedia* and *S. flavescens* were normal in morphology (Figures 7B,C). The observation of electron microscopy on section of nodules showed that the symbiosome membrane in the nodules formed by the mutant on *A. membranaceus* and *C. intermedia* were both severely disrupted, the plant cells presented plasmolysis phenomenon, and the bacteroids showed abnormal morphology (Figures 7D,E). Nevertheless, the normal symbiosome membrane and morphology of bacteroids in nodules (Figures 7C,F) induced by the mutant on *S. flavescens* may be ascribed to the extremely promiscuous property of this legume (Jiao et al., 2015).

## Discussion

### Specific genotype of *nodE* and *nodZ* for AC rhizobia

The *nodE* gene has been proved as a major determinant of the host specificity in *S. meliloti* (Bloemberg et al., 1995) and *R. leguminosarum* (Demont et al., 1993), which is responsible for the synthesis of the acyl moiety of Nod factors. In the present study the detection of highly conserved *nodE* in both the ACiM and *R. yanglingense* CCBAU01603 implying that this gene might be also the determinant for their host specificity, and that the AC plants may recognize Nod factor with same fatty acyl groups.

According to the previous research, the nodulation gene *nodZ*, which encodes a lipochitinoligosaccharide (nodulation factors) fucosyltransferase (Quinto et al., 1997), was a host-specific gene in *B. japonicum* (Stacey et al., 1994), *Sinorhizobium* sp. NGR234 (Quesadavincens et al., 1997), and *M. loti* (Rodpothong et al., 2009). In the present research, the identification of highly conserved *nodZ* in the six AC-originating *Mesorhizobium* strains and their difference from other *Mesorhizobium* strains evidenced that this gene may contribute to the host specificity in the AC rhizobia. However, the great difference between the *nodZ* genes in *R. yanglingense* CCBAU01603 and in the ACiM strains demonstrated that the AC plants might recognize *nod* factors with various molecular structures, or both *nodZ* gene types could generate the same nod factors, for which further study to estimate the nod factor patterns for both the ACiM and *R*. *yanglingense* CCBAU01603 is needed.

### Highly conserved hydrogenase systems of AC-originating strains

Hydrogen uptake that accompanying the nitrogen reduction could increase the nitrogen fixation efficiency by recycling the hydrogen for nitrogen reduction (Schubert and Evans, 1976). The hydrogenase structural and most assessor genes are only expressed under the control of the nitrogen fixation regulatory protein NifA (Brito et al., 1997; Ruizargueso et al., 2001). Thus, the hydrogenase biosynthesis was relied upon nodulation gene expression and nitrogen fixation. Hence, there may be some relationships between the hydrogenase system and host specificity preference in the AC rhizobia. Previously, hydrogen oxidation ability was discovered in *Bradyrhizobium* sp. (*Lupines*) (Murillo et al., 1989), *Bradyrhizobium* sp. (*Vigna*) (Baginsky et al., 2005), a few strains in *R*. *leguminosarum* bv. *viciae* (Ruiz-Argüeso et al., 1978; Fernandez et al., 2005) and *Rhizobium tropici* (Van Berkum et al., 1994), but was never reported in *Mesorhizobium*. To our best knowledge, hydrogenase systems were common in *Bradyrhizobium*, but rarely identified in *Mesorhizobium, Rhizobium*, and *Sinorhizobium* (Baginsky et al., 2002). Hence, the prevalent conservation of hydrogenase biosynthesis system in the AC rhizobia (including *Mesorhizobium* and *Rhizobium*) may be an environmental or host adaptation strategy. As reported in previous research, pivotal and conserved hydrogenase genes *hupS* and *hupL* showed great divergence among the *Rhizobium* and *Bradyrhizobium* strains, implying that their hydrogenase systems may be obtained from different sources or have evolved independently (Baginsky et al., 2002). But, in our study, although the hydrogenase systems prevalently exist in ACiM strains evolved distinctively from *Bradyrhizobium* and *Rhizobium*, which representing a novel lineage of hydrogenase system, but the hydrogenase system of *M. caraganae* CCBAU01502 evolved divergently from other *Mesorhizobium* strains, and keep high homology with *R. yanglingense* CCBAU01603 and other *Rhizobium* strains. These results indicates that the hydrogenase system does not stably coevolve with house-keeping core genes, and could horizontally transfer among different genera, and the existence of hydrogenase in the AC rhizobia may not related to their host specificity, but to the nitrogen fixation efficiency.

### Highly conserved *nodO* genes in AC rhizobia

The *nodO* gene is partially homologous to the hemolysin gene in *E. coli* and it located at the downstream of *nodDEFDABCIJZ* gene cluster. Similar to hemolysin, NodO protein could be secreted on the growth medium independent on the flavonoids synthesis (Scheu et al., 1992; Sutton et al., 1996) by the type I secretion system (Fauvart and Michiels, 2008), but some study showed that NodO was only produced when bacteria grew in the presence of a flavonoid *nod* gene inducer (Russo et al., 2006). Anyway, *nodO* was defined as a mutual feature that shared among ACiM and strain *R. yanglingense* CCBAU01603, and also defined in 14 representative strains of 367 isolates from *Astragalus*, being a common feature for AC rhizobia. Up to now, *nodO* has only been reported in *R. leguminosarum* bv. *viciae* and is proved responsible for host range of vetch, *Leucaena leucocephala, Phaseolus vulgaris*, and *Trifolium repens*. Moreover, the heterologous expression of *nodO* could extend the host range of rhizobia (Downie and Surin, 1990; Van Rhijn et al., 1996; Vlassak et al., 1998) and enable *R. leguminosarum* bv. *trifolii* to nodulate vetch (Economou et al., 1994). Also, *nodO* gene has been identified as nodulation and host-specific recognition factor of rhizobia associated with pea and *Vicia* spp. in *R. leguminosarum* bv. *viciae* (Economou et al., 1990; Scheu et al., 1992). Indeed, the *nodO* gene in *R. leguminosarum* bv. *viciae* encodes a hemolysin homologous ${\text{Ca}}_{2}^{+}$-binding protein without N-terminal cleavage (Economou et al., 1990), and NodO protein could trigger cation-selective channels that allow K^+^ and Na^+^ across the cell membranes. Therefore, NodO may facilitate the uptake of *nod* factors or function synergistically with depolarization or complete the deficiencies in Nod factor signaling (Sutton et al., 1994). Hence, *nodO* is presumed to participate in recognition and signal transduction for rhizobia to nodulate AC plants. Interestingly, this estimation related two previous reports together, *Neorhizobium galegae* that could nodulate *Astragalus* plants (Zhao et al., 2008) also contained a *nodO* gene (Osterman et al., 2015). Although, a unique lineage of *nodO* was detected in all the AC-nodulating strains but failed in the other strains in our study, this gene might not be directly related to the host specificity according to the nodulation tests with the *R. yanglingense* CCBAU01603 *nodO* mutant. Nevertheless, the deletion of *nodO* gene affected the development of bacteroids in nodules of the AC plants, indicating that *nodO* has an impact on the nodule formation process, but not the recognition process.

### Highly conserved T1SS in AC rhizobia

Previous studies have proved that various protein secretion systems were used in rhizobia to transport effector proteins involved in nodulation process, and *nodO* was secreted via type I secretion system (T1SS) (Russo et al., 2006). T1SS could secrete proteins from the bacterial cytoplasm to extracellular environment (Salmond, 1994), and was responsible for the secretion of various toxins, lipases, and proteases (Lenders et al., 2015) in many Gram-negative bacteria (Thomas et al., 2014). T1SS has been verified to be responsible in secretion of NodO and other homologous proteins of hemolysin without N-terminal cleavage (Economou et al., 1990; Scheu et al., 1992). In our present research, 4–8 pairs of T1SS structural genes *prsD*/*prsE* were detected in 7 AC rhizobia. Out of which, we found that a pair of *prsD*/*prsE* that maintained high conservation among AC rhizobia (96.4–100% similarity) located rightly besides the conserved *nodO* gene. This pair of *prsD*/*prsE* genes in AC rhizobia kept low similarity (55.0–70.8%) with other non AC rhizobia, which indicated that the AC rhizobia has a specific T1SS system for NodO. The adjacent location of *prsD*/*prsE* genes to *nodE, nodO*, and hydrogenase system in the AC rhizobia implied that *nodO* gene in AC-isolated strains is coevolved with its adjacent T1SS, and NodO protein may be secreted specifically through the nearby *prsD*/*prsE* secretion system at downstream.

It is well-known that T3SS plays a significant role in biochemical cross-talk between bacteria (including animal- and plant-pathogen) and eukaryotic hosts (Tseng et al., 2009). However, it had been supposed to be dispensable for nodulation of rhizobia (Mazurier et al., 2006), and it may positively or negatively affect the nodulation process (Okazaki et al., 2010; Kim and Krishnan, 2013). With the development of sequencing technology, rhizobial nodulation without T3SS was found increasing (Okazaki et al., 2016). In rhizobia, the rhc gene clusters that encode T3SS-related components (Viprey et al., 1998) could be divided into three subtypes: rhc-I in *S. fredii* NGR234 (Viprey et al., 1998; Schmeisser et al., 2009) and *B. japonicum* USDA110 (Kaneko et al., 2002); rhc-II in *S. fredii* NGR234 (Tampakaki, 2014); and rhc-III in *R. etli* CFN42 (González et al., 2006; Tampakaki, 2014). Since rhc-II type T3SS was identified in 3/7 AC-nodulating rhizobial strains and non-AC-nodulating strain *S. fredii* NGR234 (Pueppke and Broughton, 1999), while atypical T3SS was detected in the other four AC-nodulating rhizobial strains, it might be estimated that the rhc-II type T3SS may not be involved in the specific nodulation on AC plants. Moreover, the finding of atypical T3SS lineages noted as flagellar synthesis genes (annotated using the Non-Redundant Protein Database) in the 4 AC rhizobia demonstrated the possibility that the flagellar synthesis serves a function as a novel type of T3SS for AC rhizobia.

## Conclusions

Comparative genomics revealed that a large conserved fragment of functional gene clusters including evolutionarily conserved *nodE, nodO*, T1SS, and hydrogenase system were detected in the *Astragalus*/*Caragana*-associating *Mesorhizobium* and *Rhizobium* strains, and the *nodO* gene was found prevalently exists in common AC rhizobia. These genes were deduced to participate host recognition of AC rhizobia. More profoundly, the existence of particular T1SS is inferred as a factor to regulate the host specificity in these strains. These results revealed that there exist several extremely relevant genomic influencing factors for the preference between host and their rhizobia, and provided substantial materials for further research on the specificity of symbiosis between rhizobia and legumes at the cross-nodulation level.

## Availability of data and materials online

The datasets originally sequencing and analyzed during the current study are available in the National Center for Biotechnology Information (NCBI):

CCBAU01550 (https://www.ncbi.nlm.nih.gov/bioproject/243004);

CCBAU01570 (https://www.ncbi.nlm.nih.gov/bioproject/243002);

CCBAU01399 (https://www.ncbi.nlm.nih.gov/bioproject/235359);

CCBAU45272 (https://www.ncbi.nlm.nih.gov/bioproject/?term=CCBAU%2045272);

CCBAU01502 (https://www.ncbi.nlm.nih.gov/bioproject/?term=CCBAU%2001502);

CCBAU01603 (https://www.ncbi.nlm.nih.gov/bioproject/?term=CCBAU+01603);

CCBAU01583 (https://www.ncbi.nlm.nih.gov/bioproject/?term=CCBAU+01583).

All data analyzed during this study are included in this published article and its additional files.

## Author contributions

WFC and ZLD conceived and designed this research. HY performed the experiments. HY, JBX, SKJ, and NY performed the data analysis. CFT helped to improve to analysis. HY and ETW wrote this manuscript. ZJJ, LZ, ZYW, and WXC helped improving the manuscript. All authors read and approved the final manuscript.

### Conflict of interest statement

The authors declare that the research was conducted in the absence of any commercial or financial relationships that could be construed as a potential conflict of interest.
