# Supplementary material for: Evolutionarily Conserved nodE, nodO, T1SS, and Hydrogenase System in Rhizobia of Astragalus membranaceus and Caragana intermedia
Source: Front Microbiol. 2017 Nov 20;8:2282. doi: 10.3389/fmicb.2017.02282 (PMC5702008; doi:10.3389/fmicb.2017.02282)
Supplement: Supplementary file 1 [file Table1.docx]

**Supplementary Material to:**

Yan H. et al. Evolutionarily conserved *nodE*, *nodO*, T1SS and hydrogenase system in rhizobia of *Astragalus membranaceus* and *Caragana intermedia*. Frontiers in Microbiology.

**Table S1. Nodulation and amplification of *nodO* gene from strains isolated from *A. membranaceus* or *A. mongholicus*.**

| **Strains (CCBAU No.)** | **Nodulation capability** | **Amplification of *nodO*** |  |
| --- | --- | --- | --- |
| 75179, 03611, 03605, 73254, 75138, 75133, 03603, 75206, 75220, 03535, 03524, 75238, 75213, 73204 | + | + | |
| 75228, 73231, 75219, 75237, 03532, 03529, 73239, 75233, 75168, 75186, 75229, 73262, 75204 | – | – | |
